# Step by Step Golgi-Cox Staining for Cryosection

**DOI:** 10.3389/fnana.2019.00062

**Published:** 2019-06-19

**Authors:** Feng Zhong, Lei Liu, Jia-Li Wei, Ru-Ping Dai

**Affiliations:** ^1^Department of Anesthesiology, The Second Xiangya Hospital, Central South University, Changsha, China; ^2^Department of Anesthesiology, Guangdong Provincial People’s Hospital, Guangzhou, China; ^3^Department of Anesthesiology, Changsha Central Hospital, Changsha, China

**Keywords:** Golgi staining, neuronal morphology, dendrites, spines, cryosection

## Abstract

Golgi staining, though invented hundreds of years ago, is still a reliable method to study the cytoarchitecture of the brain. Almost all published Golgi staining protocols and methods were used for microtome, and rarely applied in cryosection, which restricted the application of this technique. Currently, several commercial Golgi-stain kits are available for both vibratome section and cryosection, but these kits are costly, and it is still challenging for researchers to obtain significant results. In the present study, we described a protocol of Golgi-Cox Staining for Cryosection, with the modified cryosection protection solution based on the Golgi-Cox method, which makes cryosection easy to apply in the section of the Golgi-Cox impregnated tissue. Our methods provide a low-cost and simple option for Golgi staining, and it will facilitate researchers to obtain useful Golgi staining results for neuronal architecture studies.

## Introduction

Golgi Staining, also called “black reaction,” was first discovered by accident (Camillo, 1873, 1879). This method could selectively visualize the entire architecture of a neuron with a clear background that thousands of neurons next to it unstained, making it possible to investigate the neuronal morphology under a microscope. Though many modifications from the original Golgi method such as the Rapid Golgi method, the Golgi–Kopsch method and the Golgi-Cox method, have been developed (Cox, 1891; Kemali, 1976; Riley, 1979; Yuste, 2015), the Golgi-Cox staining method is most widely used owing to its convenience and reliable results that it yields. Chromium salts which bind to proteins in the neuron were randomly formed during the impregnation, then transformed to black mercuric sulfide deposits upon alkali treatment (Ramón-Moliner, 1970; Špaček, 1989; Rosoklija et al., 2014). The architecture of the impregnated neuron, including cell somas, axons, dendrites and spines, could be easily visualized. However, available protocols were designed for microtomes including sliding microtomes and vibratomes (Gibb and Kolb, 1998; Zaqout and Kaindl, 2016). There are commercial kits that exist that have brief guidance for cryosections, but are expensive and do not include open accessible formulas. Therefore, it is still necessary to have easy and available Golgi-Cox staining protocols for researchers. Here, we describe a step by step novel Golgi-Cox staining method for cryosection, which is similar to the commercial Golgi staining kits; using this method will be easier to obtain a stable and personalized result in less time and with fewer reagents.

## Materials and Equipments

### Animals

Male Sprague-Dawley rats (8 weeks old; weight, 200 ± 20 g) were purchased from Hunan SJA Laboratory Animal Co. Limited (Changsha, China). All of the procedures were approved by the Institution of Animal Care and Use Committee of The Second Xiangya Hospital and Use Committee, which conformed to the Guide for the Care and Use of Laboratory Animals.

### Gelatin-Coated Slide Preparation

Gelatin solution was prepared by adding 10 g gelatin (Sigma-Aldrich, catalog number: G7041) in 1,000 ml double distilled water (DW), which was constantly stirred and heated until the gelatin was dissolved. One gram chromium potassium sulfate [CrK(SO_4_)_2_·12H_2_O; Sinopharm; catalog number: 20015260] was added into the solution and continuously stirred. Then we filtered the solution with filter paper. Dipped the clean slides in the rack into the solution for 10 s avoiding any air bubbles and subsequently placed it in the oven (65°C) overnight. The slides could be used within a month. Gelatin keeps brain sections, which are mainly made up of fat and water, from sticking to the slides. Chromium potassium sulfate provides positive ions for the slide. Hence, the brain tissue could firmly stick to the slides.

Note: it is important to use gel-coated slides, otherwise, the brain section will fall off the slide during the staining procedure! The gelatin-coated slides should be used within 3 months after preparation; otherwise, the section might crack after mounting on the slide while drying.

### Impregnation Solution Preparation

Three stock solutions are prepared as follows:

- Solution A: a 5% solution of Potassium dichromate (K_2_Cr_2_O_7_; Sinopharm, catalog number: 10016618) in 100 ml DW
- Solution B: a 5% solution of Mercuric chloride (HgCl_2_; Sinopharm, catalog number: 10013616) in 100 ml DW
- Solution C: a 5% solution of Potassium chromate (K_2_CrO_4_; Sinopharm, catalog number: 10016418) in 80 ml DW

These solutions should be dissolved and stirred. Heating is needed when preparing solution B. The stock solution should be kept in the dark for a few months. Mix 5 vol. parts of solution A, 5 vol. parts of solution B, 4 vol. parts of solution C and 10 vol. of DW by stirring them. After sufficiently mixing solutions, the working solution should be kept in the dark at least for 24 h, during which time reddish precipitates form. Remove the precipitates with a filter paper.

A total of 20 ml working solution is required for each rat brain sample. The volume of working solution depends on the number of tissues, and the working solution could be used until a week after the preparation. Metal instruments should be avoided during the preparation. Tissues treated with Golgi-Cox solution should be protected from exposure of light whenever possible.

### Cryoprotectant Solution

The cryoprotectant solution is critical to protect cryosections, including the preservation of the morphology and protecting the frozen tissue from artifacts. Hundred milliliters of a tissue-protectant solution (solution D) is prepared by dissolving the following components: 20 g sucrose (C_12_H_22_O_11_; Sinopharm, catalog number: 10021463), 15 ml glycerol (C_3_H_8_O_3_; Sinopharm, catalog number: 10010618) and the final volume is then adjusted to 100 ml with DW. Hundred milliliters cryoprotectant solution is sufficient for two rat brain samples.

### Staining Solution

For the staining step, solutions E and F are needed. Solution E (20% ammonia solution) can be made by adding 150 ml ammonia (NH_4_OH, 25.0%–28.0%; Sinopharm, catalog number: 10002108) into 50 ml DW. Solution F (1% sodium thiosulfate solution) is prepared by dissolving 3.14 g sodium thiosulfate (Na_2_S_2_O_3_·5H_2_O; Sinopharm, catalog number: 10021218) in 200 ml DW.

Note: solutions mentioned above are toxic and harmful, please perform the experiment under a fuming hood and wear protective gloves.

## Stepwise Procedures

### Tissue Harvest

After deeply anesthetized with 5% sevoflurane in the anesthesia chamber, the rat brain was harvested after cervical dislocation. Remove the pia mater carefully. Use DW to wash away the blood from the surface and separate the brain into two hemispheres for better impregnation.

### Impregnation Step

**Figure 1:**
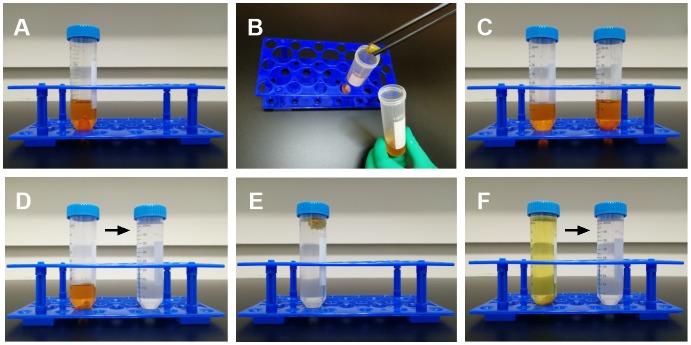
The Golgi-Cox impregnation and cryoprotection. **(A)** The rat brain was cut in a sagittal manner and immersed in the Golgi-Cox solution in the dark. **(B,C)** Tissue was transferred into fresh solution on the 2nd day, and kept at least for 2 weeks. **(D,E)** After the impregnation step, tissues were shifted to the cryoprotection solution in the dark and **(F)** the solution was replaced on the 2nd day.

Immerse the hemispheres in each centrifuge tube containing 10 ml working solution with the area of interest oriented upward (Figure 1A) and store it in a dark at room temperature. Replace the working solution after 24 h (Figures 1B,C). High or low temperatures will make neurons over- or under-stained. Two weeks is enough for impregnation. For the deep layer staining, it might need an extra week after changing the position of the tissue set in the container for better penetration.

### Cryoprotection Step

Use plastic forceps to pick out the tissue, and subsequently wipe the working solution away with tissue paper. Then transfer it into the cryoprotectant solution (solution D) at 4°C with gentle shaking (Figures 1D,E). The volume of cryoprotectant solution is 50 ml for two hemispheres. Replace the solution after 24 h (Figure 1F). If the tissue sinks into the bottom, you may start sectioning. It usually takes 2–3 days.

### Sectioning Step

**Figure 2:**
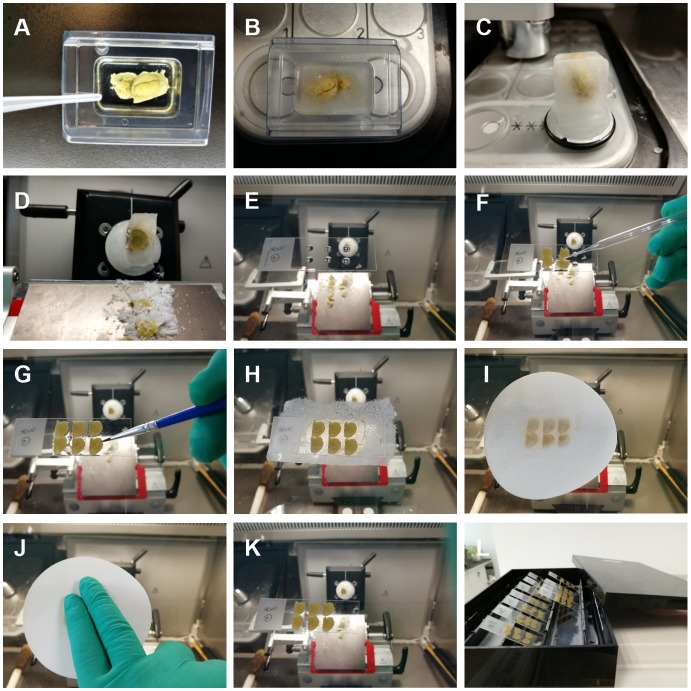
Brain sectioning. **(A–C)** The impregnated tissue was mounted with distilled water (DW) in mold and frozen in the cryostat. **(D–F)** After being sectioned, the slice was transferred by a retriever onto a gelatin-coated slide with few drops of sucrose solution on it. **(G,H)** We used a fine paint brush to adjust the position of the brain section and carefully covered it with a piece of lens cleaning tissue so that the section did not moved. **(I,J)** A filter paper was applied above the lens cleaning tissue to absorb the excess solution, and we pressed the paper to make sure any excessive solution was absorbed and no air bubbles were left between the section and the slides. **(K,L)** Move the filter paper and lens cleaning tissue away and be careful not to alter the position of the sections. Put the sections in a rack at room temperature overnight until they are fully dried.

In our methods, cryostat was used for tissue sectioning and shown in Figure 2.

Step A: The temperature of the cryostat (CM1950, Leica Biosystems) should be −20°C to −25°C. Embed the tissue in the mold with DW.

Step B: Freeze the tissue in the cryostat for 10 min.

Step C: Add a few drops of DW on the chuck to form an ice basement. Mount the brain tissue on the basement, add more DW on the tissue and wait for it to freeze and adhere to the chuck.

Step D: Section the brain with the cryostat; 100–200 μm section is adequate for dendritic arborization and spine analysis. Use a paintbrush to separate the brain section if it is sticky.

Step E: Add a few drops of 30% sucrose solution to the slide with Pasteur pipette, sucrose solution makes the brain slice adhered to the slide firmly.

Step F: Transfer the section with a glass specimen retriever onto the slide, use a fine paint brush to adjust the position of the section and let it be flat. Use a filter paper to remove excessive sucrose solution around the sections.

Step G: Adjust the orientation of the section on the slide with the brush.

Step H: Use a lens cleaning paper (GE Healthcare, Chicago, IL, USA, catalog number:10149470) to cover the section for protecting it from damage or any movement.

Step I: A piece of filter paper is used to absorb the excessive solution.

Step J: A gentle pressure is applied on the filter paper with fingers. Make sure that there is no air bubbles or solution between the section and the slide. This step is important to prevent the brain section from falling off from the slide.

Step K: Remove the lens cleaning tissue carefully.

Step L: Put the sections mounted slides in the racks and keep them in a dark room to dry overnight.

### Staining Procedure

Wash the slides with DW twice for 5 min each. Then incubate the slides in 20% ammonia solution for 10 min in the dark by gently shaking them. Wash the slides with DW twice for 5 min each. Rinse the section in 1% sodium thiosulfate solution and gently shaking it for 10 min. Wash the sections with DW twice for 5 min each. Sections can be counterstained with 1% cresyl violet if needed.

### Dehydration and Mounting Step

Dehydrate the section by processing the slides through 50%, 75%, 95%, and 100% (twice) ethanol for 5 min each. Defat the section with xylene twice for 5 min each. Then use permount Mounting Medium (Fisher Scientific, Waltham, MA, USA, catalog number: SP15-100) to mount the slide and keep it in a dark room until microscopic examination.

### Image Acquisition and Analysis

The image was obtained by optical microscopy (BX53, Olympus Corporation, Tokyo, Japan). Dendrite branches were traced by ImageJ software (NIH, Bethesda, MD, USA) with the NeuronJ plugin (Meijering et al., 2004), and the dendritic length and number of the branches were calculated. Neuronal arborization was analyzed by counting the number of crossing by dendrites of concentric circles originating at the soma using the sholl analysis plugin in ImageJ (Ferreira et al., 2014). The dendritic spines were counted by ImageJ according to the methods previously reported (Risher et al., 2014).

## Results and Discussion

Golgi-Cox staining is a useful method for visualizing the dendritic branching pattern and dendritic spines, which allows us to study the relationship between behavioral phenotype and morphological changes of neurons. In most published methods of Golgi-Cox staining, vibratome is needed for sectioning (Das et al., 2013; Zaqout and Kaindl, 2016). However, in numerous labs, cryostat is more commonly used than vibratome. The use of cryostat sectioning for Golgi staining will facilitate the researcher to have a fine section comparatively. Moreover, after cryoprotection step, the tissue could be stored at −70°C for a few months. It allows researchers to make maximum use of valuable samples and saves time, after all, it will take half a month to impregnate the tissue. Currently, there are two commercial kits for cryostat section (FD Rapid GolgiStain Kit, FD Neurotechnologies; Hito Golgi-Cox OptimStain^TM^ Kit). The copyright and confidential formula of the solution and reagents in these commercial kits will impede its widespread use in the anatomy and histology labs. In the present study, we developed an easier and more convenient protocol, which could be applied in cryostat sectioning. The Golgi solution is prepared in a short time, and the reagents and chemicals are available and less expensive. Our protocol will provide a reliable and easy way to carry out this experiment compared to using the kit based assay.

**Figure 3:**
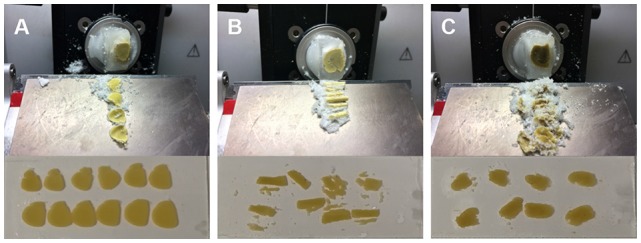
Section and mounting conditions on the slide after impregnation with different cryoprotection solutions. **(A)** Using the method mentioned in this protocol provided intact and smooth slices. **(B)** Following immersion of brain in 30% sucrose solution consequenced into porous and rigid slices and which can easily be cracked. **(C)** Cryoprotection solution reported previously (Zaqout and Kaindl, 2016) turn the brain tissues into soft and sticky mass and hard to get intact slices. The mounted slices clearly show the difference for the cryoprotection methods **(A)** from the rest of two methods **(B,C)** integrated below respectively.

Tissue protectants are crucial for cryosection. As shown in Figure 3A, complete and smooth brain slices can be easily obtained by using our methods for cryosection. However, the 30% sucrose solution or the protectant reported previously (Zaqout and Kaindl, 2016) were applied, both failed to preserve normal structures during cryosection (Figures 3B,C). However, in our methods, we did achieve complete and fine sections with the proper mixture of glycerol and sucrose solution. Glycerol softens the tissue and protects it from cracking during sectioning.

**Figure 4:**
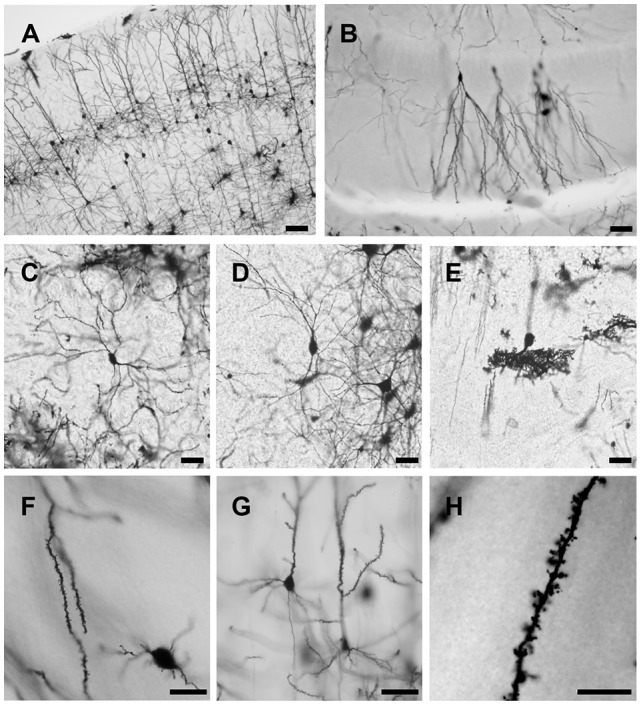
Representative images of Golgi-Cox impregnated brain slice of adult rats. Pyramidal neurons in cortex **(A)**, granule cells in hippocampus **(B)**, pyramidal neurons in striatum **(C)** and thalamus **(D)** and Purkinje cells in cerebellum **(E)** are well stained. Spines can be observed along the dendrite **(F,G)** even counted at higher magnification **(H)**. Scale bars 100 μm in **(A)**, 50 μm in **(B–E)**, 20 μm in **(F,G)** and 10 μm in **(H)**.

**Figure 5:**
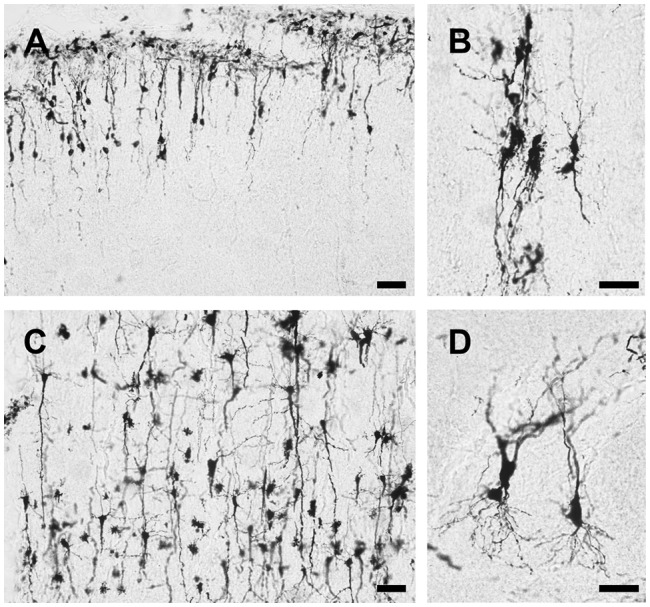
Representative images of Golgi-Cox impregnated brain slice of young rats. Cortical neurons were stained in PND0 **(A)** and PND7 **(C)** rat brain using our methods. Pyramidal neurons stained in PND0 and PND7 rat’s brain shown at higher magnification in **(B,D)** respectively. Scale bars 50 μm in **(A–D)**.

Using this method, both the dendritic tree and spines of neuron are fine stained in the cortex, hippocampus and other deep layer brain regions striatum, thalamus and cerebellum (Figure 4). The stained sections did not show fading; rather, they remained clear and drawable even 2 years after staining. Moreover, our methods also could be applied on the developing brain, though less number of neurons were stained in Postnatal Day 0 (PND0) and PND7 compared to adult brain (Figure 5). Our methods provide a simple protocol to perform Golgi staining with less chemical use and high efficiency. Taken together, our protocol has established a complete procedure from solution preparation to staining with details to examine the morphology of neuronal dendrites and spines.

## Ethics Statement

All of the procedures were approved by the Institution of Animal Care and Use Committee of The Second Xiangya Hospital and Use Committee, and conformed to the Guide for the Care and Use of Laboratory Animals.

## Author Contributions

FZ and LL conducted the study, manuscript writing and revision. J-LW helped in performing the experiment. R-PD designed and interpreted experimental results, prepared the manuscript drafting and revision. All authors approved the final version of the manuscript.

## Conflict of Interest Statement

The authors declare that the research was conducted in the absence of any commercial or financial relationships that could be construed as a potential conflict of interest.
